# Herpesvirus-host interactions in neurological diseases: the immunogenetic role of HLA-E

**DOI:** 10.1128/jvi.00869-25

**Published:** 2025-11-24

**Authors:** Marianne Graninger, Elisabeth Puchhammer-Stöckl, Hannes Vietzen

**Affiliations:** 1 Center for Virology, Medical University of Vienna 27271, Vienna, Austria; Universiteit Gent, Merelbeke, Belgium

**Keywords:** human herpesviruses, HLA, Epstein–Barr virus, cytomegalovirus, herpes simplex virus, encephalitis, multiple sclerosis, natural killer cells, varicella-zoster virus

## Abstract

Human herpesviruses (HHVs) comprise nine pathogenic members, including herpes simplex virus 1, herpes simplex virus 2, varicella-zoster virus, Epstein–Barr virus, human cytomegalovirus, human herpesvirus 6A/B, human herpesvirus 7, and Kaposi’s sarcoma-associated herpesvirus. Clinical manifestations of HHV infection can range from asymptomatic cases to a broad spectrum of neurological complications, spanning from acute conditions such as encephalitis to chronic disorders including Alzheimer’s disease and multiple sclerosis. By establishing latency and undergoing repeated reactivation, HHVs maintain lifelong interactions with the human immune system and shape host immune responses, exerting considerable impact on nervous system homeostasis. Individual susceptibility to, and outcomes of, HHV-associated neurological disorders depend on multiple factors, including the infecting HHV strain and host genetics. Recent evidence highlights the pivotal role of the human leukocyte antigen E (HLA-E) pathway—a non-classical major histocompatibility complex class I molecule with immunomodulatory functions—in regulating virus–host interactions. Since some HHVs manipulate HLA-E to evade immune recognition, individual variability in this axis may influence neurological outcomes. In this review, we summarize and discuss current knowledge of the role of HLA-E in herpesvirus-associated neurological diseases.

## HUMAN HERPESVIRUSES AND NEUROLOGICAL DISEASES

The human herpesvirus (HHV) family comprises nine pathogenic members: herpes simplex virus 1 (HSV-1), herpes simplex virus 2 (HSV-2), varicella-zoster virus (VZV), Epstein–Barr virus (EBV), human cytomegalovirus (HCMV), human herpesvirus 6A (HHV-6A), human herpesvirus 6B (HHV-6B), human herpesvirus 7 (HHV-7), and Kaposi’s sarcoma-associated herpesvirus (KSHV) (1). HHV infections may contribute to the development of both (sub)acute and chronic neurological diseases, which result from nervous tissue damage either in the scope of lytic viral replication or from extensive virus-directed or autoreactive immune responses. An overview of the neurological complications of HHV infections is provided in Table 1.

**TABLE 1:** Overview of neurological complications of human herpesvirus infections

Human herpesvirus	Associated acute and subacute neurological diseases	Associated chronic neurological disorders	References
Herpes simplex virus 1	Encephalitis, meningitis, acute retinal necrosis; post-infectious autoimmune encephalitis	Putative role in Alzheimer’s disease, possible role in Parkinson’s disease	2 – 5
Herpes simplex virus 2	Meningitis (single episode or recurrent); encephalitis (particularly in neonates or immunocompromised individuals)	Possible role in Alzheimer’s disease	6, 7
Varicella-zoster virus	Meningitis, encephalitis, cerebellitis, cranial nerve palsies, myelitis, radiculitis, acute retinal necrosis, vasculopathy	Congenital VZV (e.g., microcephaly, cortical atrophy, seizures); postherpetic neuralgia; increased risk of dementia and Parkinson’s disease	2, 8–12
Epstein–Barr virus	Meningitis, encephalitis, myelitis; polyradiculopathy, acute cerebellar ataxia; acute disseminated encephalomyelitis	Strongly associated with multiple sclerosis; possible role in Alzheimer’s disease and Parkinson’s disease	13 – 16
Human cytomegalovirus	Meningitis, encephalitis, polyradiculopathy, polyneuropathy	Congenital CMV (e.g., cerebral calcification, microcephaly, motor impairments, sensorineural hearing loss); possible role in Alzheimer’s disease, Parkinson’s disease, controversial role in multiple sclerosis	17 – 20
Human herpesvirus 6A	Unclear	Possible role in Alzheimer’s disease and multiple sclerosis	21 – 23
Human herpesvirus 6B	Encephalitis (primarily children and immunocompromised individuals)	Possible role in Alzheimer’s disease and multiple sclerosis	21 – 25
Human herpesvirus 7	Unclear	Possible role in Alzheimer’s disease	21
Kaposi’s sarcoma-associated herpesvirus	Meningoencephalitis (rarely, immunocompromised patients)	Unclear	26

Notably, clinical manifestations of HHV infections range from asymptomatic infection to severe central nervous system (CNS) inflammation with long-term neurological sequelae and progressive neurodegenerative syndromes (27, 28). Central to this variability in disease susceptibility and outcome is the complex interplay between the infecting virus and host immune responses. Owing to their ability to establish latent infection and undergo recurrent reactivation, HHVs exert a lifelong influence on the host immune system (29). The long-term interplay between herpesviruses and intrinsic, innate, and adaptive immune pathways shapes host immune functions with significant implications for nervous system homeostasis. Coinfection with multiple herpesviruses further increases the complexity of virus–host interactions and complicates the investigation of disease associations. Moreover, the risk of developing herpesvirus-related neurological disease depends heavily on individual factors, including the specific viral strain, timing of infection, and host genetic predispositions that influence immune responses and susceptibility to nervous system infection.

In this context, recent research has underscored the importance of the human leukocyte antigen E (HLA-E) pathway. HLA-E is a non-classical major histocompatibility complex (MHC) class I molecule with key immunomodulatory functions, responsible for fine-tuning immune responses by balancing efficient viral control with the prevention of excessive immunopathology (30). Importantly, HHVs have evolved strategies to manipulate HLA-E expression and peptide presentation, thereby facilitating immune evasion. Variability in HLA-E expression, peptide-binding capacity, and receptor engagement could therefore influence individual susceptibility to both acute and long-term neurological complications of HHV infections.

In light of recent advances in understanding HHV-related neuroimmunology, the HLA-E axis emerges as a promising focal point for research. In this review, we therefore aim to summarize and discuss current knowledge and future directions regarding the role of HLA-E in herpesvirus-associated neurological diseases, highlighting its potential as both a biomarker and a therapeutic target.

## HUMAN HERPESVIRUS INFECTIONS AND ACUTE NEUROLOGICAL DISEASES

Acute and subacute neurological manifestations of HHV infections are well documented and include a wide spectrum of central and peripheral nervous system disorders (2, 13, 17, 25). HSV-1, HSV-2, and VZV are neurotropic viruses that establish latency in neuronal ganglia following primary infection and are most frequently associated with acute neurological diseases among the nine HHVs (2, 31). Acute inflammatory neurological diseases may arise during either primary infection or viral reactivation. Although HSV-1, HSV-2, and VZV all belong to the *Alphaherpesvirinae* subfamily, their pathogenesis in the nervous system differs substantially.

HSV-1 infection of the CNS typically results in acute encephalitis, most commonly affecting the temporal and frontal lobes, following viral entry via the olfactory nerve or anterograde transport from the trigeminal ganglia (32–34). Patients may present with fever, impaired consciousness, and focal neurological signs (31, 35). HSV-1 encephalitis (HSE) may occur at any age, but shows two incidence peaks: in children and adolescents, and in adults over 50 years, possibly representing diseases caused by primary HSV-1 infection and by viral reactivation, respectively (31, 36, 37).

The pathogenesis of HSE involves destruction of brain parenchyma due to both lytic viral replication in neurons and glial cells as well as excessive inflammatory and cytotoxic immune responses by resident and infiltrating immune cells (38–40). Despite specific antiviral treatment, HSE-related mortality can still reach up to 25% in the acute phase, and many survivors experience long-term neurological sequelae, such as cognitive impairment, speech and memory disorder, motor and sensory residuals, or seizures (41). As a subacute complication, HSE can trigger autoimmune encephalitis that may manifest with psychiatric symptoms, decreased levels of consciousness, motor impairments, and/or seizures (3).

In contrast to HSV-1, CNS infection with HSV-2 is more often associated with meningitis—either as a single or recurrent episode—in immunocompetent adults (6, 42–44). It has been suggested that insufficient HSV-2 immune control in peripheral ganglia allows replication and release of viral particles into the cerebrospinal fluid (CSF), ultimately leading to meningeal inflammation (45, 46). Furthermore, analyses of inflammatory responses within the CNS revealed a differential and more pronounced intrathecal chemokine production in the context of HSV-2 meningitis compared to HSV-1 encephalitis, possibly underlying its milder clinical course (47). However, HSV-2 may also cause encephalitis, which particularly affects, but is not limited to, neonates and immunocompromised patients (6, 42, 44, 48).

VZV infection can result in a wide range of acute neurological complications during primary infection as well as reactivation, including meningitis, encephalitis, cerebellitis, cranial nerve palsy, retinal necrosis, vasculopathy, myelitis, and radiculitis, consistent with VZV latency in spinal nerve ganglia (2, 8). VZV infection *in utero* is rare, but if symptomatic, it can lead to a variety of long-term neurological sequelae (12). VZV reactivation in spinal ganglia typically presents as herpes zoster with herpetic lesions and radicular pain but may also manifest without rash as so-called zoster sine herpete (2, 49). As a chronic complication, sensitization of nociceptors and damage to spinal structures due to long-term inflammation can result in postherpetic neuralgia in a significant proportion of herpes zoster patients (50, 51).

Beyond *Alphaherpesvirinae*, other HHVs can also affect the central and peripheral nervous systems. HHV-6B may cause encephalitis, particularly in children and immunosuppressed individuals, whereas the clinical significance of HHV-6A or HHV-7 infection remains unclear (24, 25). Acute CNS involvement in HCMV or EBV infection is relatively rare, but both viruses are associated with the development of subacute, immune-mediated conditions such as Guillain–Barré syndrome or acute disseminated encephalomyelitis, respectively (13, 52). Notably, HCMV is the most common congenital infection affecting 0.5%–2% of newborns and can result in severe chronic neurological impairments (17). Finally, although neurological complications due to KSHV have primarily been described in immunosuppressed individuals, direct involvement of KSHV in neurological disease remains to be determined (26).

## HUMAN HERPESVIRUS INFECTIONS AND CHRONIC NEUROLOGICAL DISORDERS

Over the past decades, growing evidence has linked HHVs to the development of chronic neurological disorders. The most prominent example is the strong epidemiological and mechanistic association between EBV and multiple sclerosis (MS), a chronic neuroinflammatory and autoimmune disease of the CNS. A recent study reported that the risk of developing MS was increased 32-fold following infection with EBV, whereas no increased risk was observed after infection with other viruses, including the similarly transmitted HCMV (14). Additionally, herpesviruses have been implicated in the pathogenesis of neurodegenerative diseases, particularly Alzheimer’s disease (AD). HSV-1 is the most extensively studied HHV in this context (4). Post-mortem analyses, *in vitro* studies, and animal and organoid models have demonstrated that HSV-1 infection is associated with amyloid β (Aβ) deposition in the brain, a hallmark of AD (53–57). These findings support the hypothesis that Aβ may have protective antiviral functions and accumulates over time as a result of repetitive, subclinical HSV-1 reactivations within the CNS (22). Similar observations have been reported for HHV-6A, HHV-6B, and HHV-7 CNS infections, but their clinical relevance remains less clear (21, 22). Emerging data suggest that HCMV, through replication in the gut and subsequent transport via the vagus nerve, may also promote Aβ accumulation in the CNS (18). VZV reactivation has likewise been linked to an increased risk of dementia, and recent studies highlight the protective effect of herpes zoster vaccination in reducing this risk (9, 10, 58), likely through the prevention of neuroinflammatory processes.

Besides AD, a role of herpesvirus infection in the development of Parkinson’s disease (PD) has been proposed. Studies have indicated the possibility of molecular mimicry between α-synuclein and HSV-1, as well as EBV-specific peptides, potentially driving autoimmune responses directed against α-synuclein deposits in dopaminergic neurons, thereby promoting neuronal degeneration in the substantia nigra (5, 16). Epidemiological data have linked herpes zoster to an increased risk of PD development (11). The role of HCMV in PD remains controversial. Some studies suggest that chronic neuroinflammation associated with HCMV-driven immunosenescence may contribute to PD progression, while a recent seroprevalence study reported an increased risk of PD in HCMV IgG-seronegative males (17, 19). Overall, the role and mechanism of HHV infection in PD remain unclear and deserve further investigation.

## HLA-E: A CONSERVED IMMUNOREGULATOR

The HLA is a highly polymorphic genomic region on chromosome 6 that encodes molecules responsible for pathogen recognition by T cells and the regulation of immune responses. HLA class I genes are broadly divided into classical (HLA-A, HLA-B, and HLA-C) and non-classical genes (HLA-E, HLA-F, HLA-G, HFE, MR1, and MHC-like proteins of the CD1 family) (59, 60). While classical HLA class I molecules are highly polymorphic, ubiquitously expressed on nucleated cells, and present a broad array of endogenous peptides to cytotoxic CD8^+^ T lymphocytes (CTLs), non-classical class I molecules, by contrast, are characterized by restricted tissue expression, limited polymorphism, and a specialized immunoregulatory role.

HLA-E is a non-classical HLA class I molecule with specific immunoregulatory functions. Evolutionary studies indicate that the HLA-E locus is the most highly conserved among all primate MHC class I genes (61). The baseline surface expression of HLA-E is low in most nucleated cells compared to classical MHC class I molecules (62). *In vitro* studies have reported that HLA-E has a short cell surface half-life, reflecting its residence time at the plasma membrane, of about 12 minutes compared to several hours for classical HLA class I molecules, after which it is rapidly internalized and trafficked from the cell surface to endosomes (60, 63, 64). There, HLA-E is presumably loaded with lysosome-digested peptides and then recycled to the cell surface (65). Aside from being present in its membrane-bound form, HLA-E can also be released through cleavage by metalloproteinases in a soluble form (sHLA-E), which is thought to protect bystander cells from excessive immune damage (66, 67).

While HLA-E transcripts are broadly distributed across tissues, high-level HLA-E protein surface expression is physiologically restricted to resting and activated leukocytes and endothelial cells (30). Under pathological conditions, such as infections or tumors, HLA-E expression is, however, often upregulated in most tissues. Studies in tumor models indicate that this increase in HLA-E is mainly driven by gene transcription mechanisms influenced by cytokines such as tumor necrosis factor α (TNFα), interleukin-1β (IL-1β), and interferon γ (IFNγ) via STAT1-dependent or via Class II transactivator/SXY regulatory pathways (67–69).

The HLA-E gene comprises seven exons encoding structural HLA-E components: exon 1 encodes the leader peptide; exons 2, 3, and 4 encode the α1, α2, and α3 domains, respectively; exon 5 encodes the transmembrane region; and exons 6 and 7 encode the cytoplasmic tail (visualized in reference 70) (67). The HLA-E molecule is assembled in the endoplasmic reticulum (ER) into a trimeric complex consisting of the heavy chain (~45 kDa), containing the α1, α2, and α3 domains, which is non-covalently bound to β₂-microglobulin and a nonameric peptide tail (visualized in reference 71) (67). HLA-E has a less flexible binding pocket than classical HLA I molecules, which limits peptide-dependent conformational changes. Peptides are bound via two main anchors (P2 and P9) and three secondary anchor residues at positions P3, P6, and P7 (60, 67). HLA-E-bound peptides are preferentially derived from signal leader sequences of other HLA class I molecules and have the canonical sequence VMAPRT(L/V)(V/L/F)L, thus excluding only a few HLA-B and HLA-C allotypes, as well as leader sequences from HLA-F and HLA-E (72, 73).

Under physiological conditions, surface-bound, peptide-loaded HLA-E engages heterodimeric CD94/NKG2A receptors expressed on distinct subsets of cytotoxic natural killer (NK), NKT, and some CD8^+^ T cells (67, 74). The interaction between HLA-E and the inhibitory CD94/NKG2A receptor delivers a strong negative signal via the ITIM motifs in the cytoplasmic domain of NKG2A, suppressing NK and T cell cytotoxicity (75, 76). This protects healthy, MHC class I-expressing cells from lysis and mediates immune tolerance to autologous cells. HLA-E-mediated immune regulation thus plays a pivotal role in maintaining self-tolerance and preventing autoimmunity.

## HLA-E IN THE CENTRAL NERVOUS SYSTEM

The CNS is considered an immunoprivileged organ, where interactions with the peripheral immune system are tightly coordinated at restrictive barrier sites, namely the blood-brain barrier (BBB) and blood-CSF barrier. The CNS harbors unique immunological features that support an immunosuppressive environment. Under physiological conditions, HLA-E expression in the CNS is relatively low compared to peripheral tissues; however, HLA-E is detectable on multiple resident cell types, including microglia, astrocytes, and the endothelial cells of the BBB (Human Protein Atlas, proteinatlas.org [77]) (78).

Recent transcriptomic analyses have shown that HLA-E is predominantly expressed in microglia, enabling these resident immune cells to participate in local immune regulation. In contrast, astrocytes show only basal levels of HLA-E expression under healthy conditions. This restricted expression pattern suggests a role of HLA-E in local immune surveillance and homeostasis within the CNS. The engagement of HLA-E with the inhibitory receptor CD94/NKG2A on CNS-resident or infiltrating NK cells and CTLs likely delivers a “do not kill” signal, preventing the lysis of healthy cells (67)—a mechanism especially critical in the CNS, where excessive immune-mediated cytotoxicity could cause irreversible damage to neurons and glial cells.

HLA-E expression on endothelial cells is thought to protect the BBB from cytotoxicity by circulating NKG2A^+^ NK and T cells. A recent study reported a reduction of HLA-E expression in endothelial cells of the BBB in amyotrophic lateral sclerosis as well as in patients with frontotemporal lobar degeneration, suggesting that downregulation of HLA-E may facilitate BBB breakdown (79). Although baseline HLA-E expression in the CNS is low, it may be upregulated in neuroinflammatory CNS disorders. Studies have shown that TNFα, IL-1β, and IFNγ upregulate cell-surface HLA-E expression on endothelial cells *in vitro* and induce the release of sHLA-E (66). This upregulation delivers inhibitory signals that protect healthy CNS tissue from bystander damage during infections or autoimmune responses, providing a neuroprotective mechanism during CNS inflammation. In this line, a recently published study demonstrated an increased HLA-E protein expression in endothelial cells of active MS lesions (80).

These diverse disease contexts underscore that HLA-E is a central immunoregulatory node in the CNS. Its expression reflects a broad immunomodulatory response to injury and inflammation in the CNS, restraining excessive immune activation while preserving neural integrity.

## HLA-E POLYMORPHISM

In comparison to the highly polymorphic classical HLA class I molecules, HLA-E exhibits remarkable conservation and low polymorphism. In the human population, HLA-E is predominantly represented by two functional alleles: HLA-E*01:01 and HLA-E0*01:03, which together comprise over 99% of alleles across diverse ethnicities, implying persistent stabilizing selection acting on HLA-E over an extended period (81, 82). Globally, HLA-E*01:01 is more frequent in Africa and the western part of South America, while HLA-E*01:03 is more common in European, Southeast Asian, and East Asian populations. Beyond these two common alleles, rare HLA-E variants exist, but they typically differ by synonymous changes or amino acid substitutions outside the peptide-binding groove, and their functional impact remains poorly understood (83).

HLA-E*01:01 and HLA-E*01:03 differ by a single nonsynonymous substitution in exon 3. This base change alters codon 107 in the α2 domain of the heavy chain, resulting in an arginine (Arg) in HLA-E*01:01 or a glycine (Gly) in HLA-E*01:03 (30, 84, 85). Amino acid 107 is located in an outwardly exposed loop below the α2-helix of the peptide-binding platform and does not affect the general structure of peptide-assembled HLA-E molecules (30, 86). HLA-E*01:03 appears to be the older allele, as Gly107 is exclusively found in all primate HLA-E orthologs (81).

Although subtle, this amino acid substitution affects several molecular and functional properties. First, expression levels differ between the alleles: HLA-E*01:03 demonstrates greater thermal stability and is generally expressed at higher steady-state levels on the cell surface compared to HLA-E*01:01, likely due to enhanced assembly efficiency with β2-microglobulin, slower ER egress, and improved peptide binding and loading. Second, peptide-binding stability is also affected: HLA-E*01:03 forms more thermally stable peptide-MHC complexes, particularly with canonical leader sequence-derived nonameric peptides from other HLA class I molecules. This increased stability extends the half-life of HLA-E*01:03 at the plasma membrane, prolonging its availability to engage with immune receptors (30, 86–91).

Functionally, high HLA-E*01:03 expression through binding of viral peptides may dampen cytotoxic responses via enhanced CD94/NKG2A engagement, delaying viral clearance. In contrast, low HLA-E*01:01 expression limits inhibitory signaling, allowing faster immune activation but increasing the risk of bystander tissue damage. In this context, HLA-E*01:03 may act as a neuroprotective factor but also as a potential facilitator of viral immune evasion. Conversely, HLA-E*01:01 may enhance early viral control but pose a higher risk of long-term neuroinflammation and tissue injury. Recently, HLA-E*01:01 has been associated with higher risk for MS, potentially suggesting that this less inhibitory allele may promote excessive immune activation and predispose individuals to immune-mediated damage in autoimmune or inflammatory CNS disorders (15).

## HLA-E AND HUMAN HERPESVIRUS INTERACTION

Over the course of co-evolution with humans, herpesviruses have developed sophisticated strategies to evade immune detection, particularly by NK cells and T cells, which are crucial for controlling viral infections (92). One such strategy involves the manipulation of HLA-E expression and function. Upon HHV infection, HLA-E can bind “self” peptides from stressed cells as well as “non-self,” viral peptides from infected cells. By modulating the HLA-E axis, HHVs exploit natural immunoregulatory mechanisms intended to prevent autoreactive damage, thereby avoiding detection and elimination by the immune system (67).

**Fig 1:**
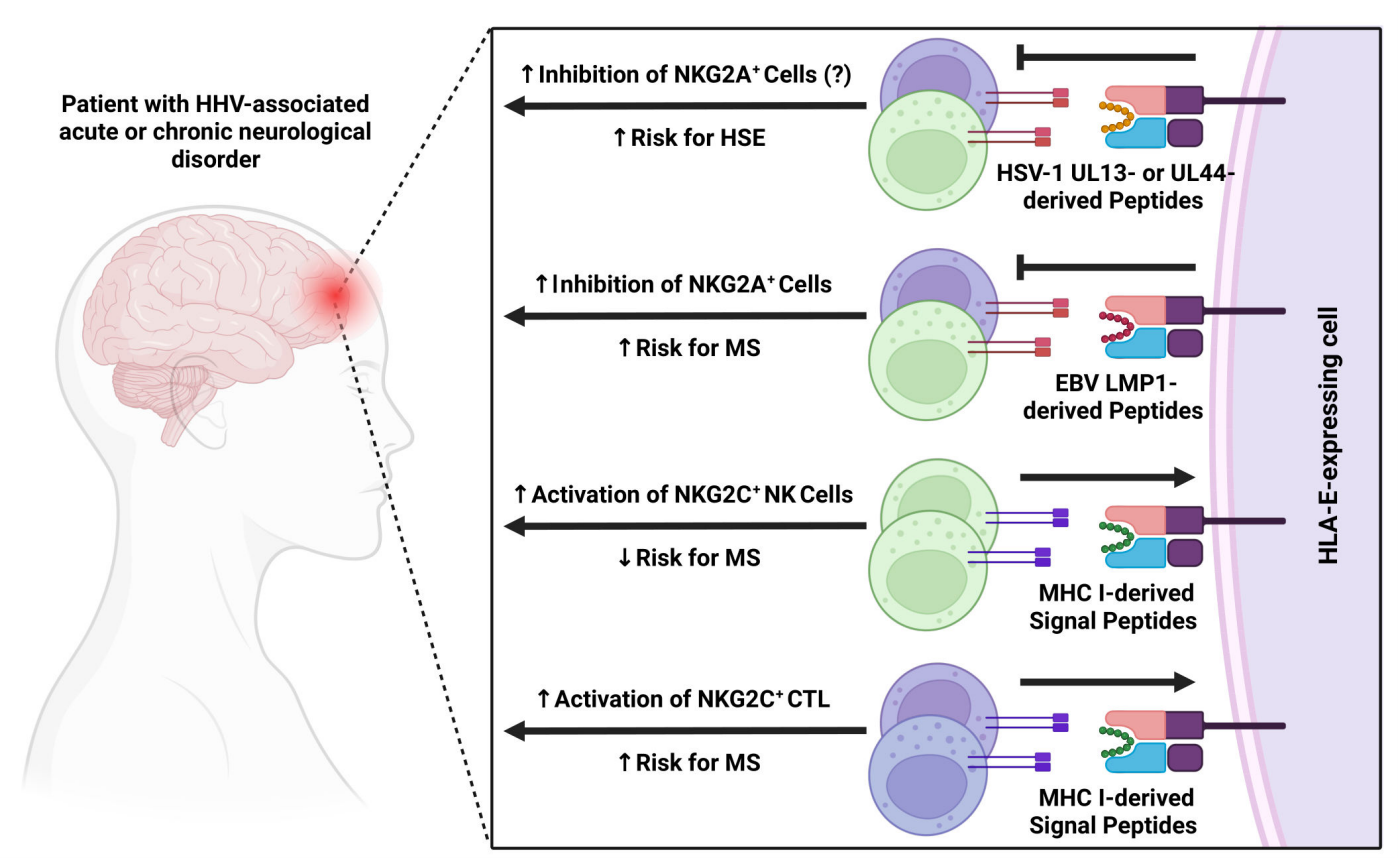
Overview of the exploitation of the HLA-E pathway by different HHVs and their impact on neural homeostasis. Green cells indicate NK cells, while purple cells indicate CTL. CTL, cytotoxic T cell; NK cell, herpes simplex virus type natural killer cells.

During acute infection, HHVs typically induce downregulation of classical MHC class I molecules on infected cells, a mechanism that prevents viral antigen presentation to CTLs (93). The lack of MHC class I leader peptides results in the downregulation of surface HLA-E and the loss of an immunological “self” signal, rendering infected cells highly susceptible to NK cell-mediated lysis. To circumvent this form of immune surveillance, HHVs have evolved mechanisms to upregulate HLA-E even in the absence of classical MHC class I molecules. Viral peptides can be loaded onto HLA-E, stabilizing it on the surface of infected cells, which delivers a potent inhibitory signal to NK and T cells via NKG2A and prevents the lysis of virus-infected cells. Additionally, the manipulation of HLA-E may serve the establishment and maintenance of viral latency in various cell types, depending on the specific HHV tropism. An overview of the exploitation of the HLA-E pathway by different HHVs, which markedly impacts host immune responses and, as a consequence, various aspects of neuronal homeostasis, is provided below and shown in Fig. 1. In addition, Table 2 lists published HHV-specific peptide sequences that can bind to and upregulate HLA-E. However, the presence of additional, as of yet undetected, HLA-E-binding peptides cannot be excluded. Notably, most studies of peptide binding to HLA-E rely on cell surface stabilization assays, in which peptides often induce only modest or borderline upregulation of HLA-E (58). The functional consequences of these interactions, both regarding the inhibition of NKG2A^+^ cells and the activation of HLA-E-restricted T cells or NKG2C^+^ cells, remain unclear and therefore warrant further investigation.

**TABLE 2:** HHV-specific peptide sequences able to upregulate HLA-E^*a*^

Virus	HLA-E-upregulating peptide sequence	Protein	Function	References
HSV	VLPRPTITM	Glycoprotein C (UL44)	NKG2A^+^ cell inhibition (?)	94, 95
	SSPSHILTL	Tegument serine/threonine protein kinase (UL13)	NKG2A^+^ cell inhibition (?)	94
EBV	GGDPHLPTL-derived variants	Latent membrane protein 1	NKG2A^+^ cell inhibition	15, 96, 97
	SQAPLPCVL	BZLF-1	HLA-E-restricted CD8^+^ T cells	98 – 100
HCMV	VMAPRTLIL-derived variants	UL40	NKG2A^+^ cell inhibition NKG2C^+^ cell activation HLA-E-restricted CD8^+^ T cells	73, 98, 99, 101

^*a*^ Question marks indicate presumed interactions with NK cell surface receptors.

## THE ROLE OF HLA-E IN HHV REACTIVATION

All human herpesviruses undergo recurrent, often subclinical reactivation from latent infection, and several environmental factors have been identified that can trigger viral reactivation (29, 102). Reactivation of latent HSV-1 in neurons, for example, can be induced by systemic physical or emotional stress, fever, microbial co-infection, ultraviolet exposure, or hormonal imbalance (102, 103). Many of these triggers activate classical signal transduction pathways, including protein kinase C, p38 kinase, c-Jun N-terminal kinase, ERK kinase, and PI3 kinase, which can alter viral and host transcriptional programs to initiate the lytic cycle (102).

Beyond these canonical pathways, the HLA-E pathway has recently emerged as an additional critical regulator of herpesvirus latency and reactivation. In HCMV infection, reactivations frequently occur in immunosuppressed transplant recipients, highlighting the role of impaired NK-cell and CD8^+^ T cell functions. Recent studies demonstrated that the HLA-E–UL40 axis has a substantial impact on the level of HCMV replication in transplant recipients. Individuals carrying the HLA-E*01:03/01:03 genotype who were infected with HCMV strains exhibiting high-affinity HLA-E-binding UL40 peptide variants showed enhanced stabilization of HLA-E on infected cells. This led to potent inhibition of NKG2A^+^ NK cells and a markedly increased risk of high-level viral reactivation (104, 105).

A similar mechanism may be present during EBV infection. EBV reactivation and the associated transition from latency to lytic replication are typically triggered by host cell stress or by B-cell differentiation into plasma cells through B-cell receptor engagement and cytokine signaling (102). However, recent studies have also reported a high prevalence of the HLA-E*01:03/01:03 genotype among patients with EBV-associated lymphomas, suggesting a link between this allele and impaired immune control of EBV-infected cells (97, 106). The same studies identified distinct EBV latent membrane protein 1 (LMP-1)-derived peptide variants that strongly upregulate HLA-E and are associated with potent inhibition of NKG2A^+^ NK cells, findings predominantly observed in EBV-associated lymphoma patients (97, 106). Interestingly, these peptide variants are also frequently detected in MS patients (15), suggesting that the HLA-E pathway may facilitate more frequent EBV reactivation, which, in turn, could increase the risk of EBV-associated autoimmune and lymphoproliferative diseases.

## EXPLOITING THE HLA-E/NKG2A AXIS: HSV-1 STRATEGY FOR PERSISTENCE

Engagement of the HLA-E–NKG2A pathway as an immune evasion mechanism has so far been identified for several HHVs, and recent observations indicate that this might also account for HSV-1. We have previously identified two conserved HSV-1 peptides that can bind and stabilize HLA-E on the infected cell’s surface, likely mediating inhibitory NKG2A signals on cytotoxic immune cells (94). Both of these peptides are expressed during the lytic HSV-1 replication cycle, indicating that HSV-1 must initiate at least some degree of replication in order to engage HLA-E when presenting the identified peptide sequences (107). Furthermore, we observed that the two HSV-1 specific peptides each showed preferential binding to one of the two HLA-E alleles, i.e., HLA-E*01:01 and HLA-E*01:03. In line with this observation, we showed that patients with HSV-1 encephalitis harbored an increased frequency of homozygous HLA-E genotype variants, potentially reflecting the increased binding affinity of the respective peptide sequences to their preferred HLA-E allele, resulting in increased HLA-E stabilization. Considering the presumed association between HSV-1 and AD development, engagement of the HLA-E pathway by HSV-1-infected CNS cells could facilitate immune evasion and enable repeated HSV-1 reactivations in the brain, leading to amyloid accumulation and initiation of neurodegeneration, particularly in genetically predisposed individuals (53, 55). Importantly, the interaction between HSV-1-specific HLA-E stabilization and the NKG2A receptor, both on NK cells and T cells invading the CNS, needs to be functionally confirmed and further investigated to establish its relevance in disease pathogenesis.

As a hallmark of *Alphaherpesvirinae*, HSV-1 establishes latency in neuronal ganglia, where viral replication is restricted by local NKG2A^+^ CTLs in a non-cytolytic manner via the expression of IFNγ and granzyme-mediated cleavage of immediate early HSV-1 replication products (108). Satellite glial cells (SGCs) surrounding neurons act as antigen-presenting cells capable of expressing MHC class I molecules, and SGCs have been proposed to protect HSV-1-infected trigeminal neurons from T cell-mediated cytotoxicity via expression of HLA-E and PD-L1 (109). In this context, the expression of (other) HLA-E-binding peptides during early or abortive replication of HSV-1—or other neurotropic HHVs—deserves further investigation.

## HLA-E–DRIVEN IMMUNE EVASION BY EBV AND ITS ROLE IN CNS AUTOIMMUNITY

Engagement of the HLA-E–NKG2A pathway as an immune evasion mechanism has also been reported for EBV, a ubiquitous herpesvirus that establishes lifelong latency in B cells (96). A central immune evasion mechanism involves the expression of EBV LMP-1-derived peptide variants that bind and upregulate surface HLA-E, thereby suppressing NKG2A^+^ NK cells and CD8^+^ T cell functions and impairing their ability to recognize and eliminate latently EBV-infected cells. Consequently, EBV escapes immune surveillance, which promotes viral latency and contributes to the virus’s long-term persistence.

The exploitation of the HLA-E–NKG2A immune evasion axis by EBV has profound implications for the development of MS. EBV infection is a major environmental risk factor for MS, and its capacity to modulate HLA-E through LMP-1 is thought to drive chronic immune activation and influence disease susceptibility as well as progression. Interestingly, LMP-1-derived peptides show a high degree of polymorphism, leading to differential interactions with HLA-E (15, 96, 97). Specific LMP-1-derived peptide variants, such as GGDPHLPTL and GGDPPLPTL, which strongly upregulate HLA-E surface expression, are predominantly found in MS patients. In contrast, LMP-1derived peptide variants that show only weak upregulation of HLA-E, i.e., GSDPHLPTL and GGDPHLPPL variants, occur more frequently in healthy controls compared to MS patients (15). This results in potent suppression of NKG2A^+^ NK cells and CD8^+^ T cells in MS patients, impairing their ability to recognize and kill EBV-infected cells. Consequently, EBV may hinder the clearance of infected and potentially autoreactive EBV-infected B cells, facilitating the breakdown of immune self-tolerance and fostering sustained neuroinflammation in MS. Thus, while the HLA-E–NKG2A axis normally serves to protect host tissues from immune-mediated damage, its exploitation by EBV undermines effective immune control and may set the stage for autoimmune pathology.

The strong association of LMP-1 peptide variants with the development of MS further raises the question of whether analyzing LMP-1 peptide variants together with HLA-E alleles represents a suitable strategy to identify patients at risk for MS. A recent retrospective study demonstrated a 260-fold increase in risk among individuals carrying both LMP-1 and HLA-E risk variants, suggesting that this combined analysis is promising for stratifying individual risk (15).

## EBV-SPECIFIC, HLA-E-RESTRICTED T CELLS AND MS

Beyond its canonical role in NKG2A^+^ NK and T cell regulation, HLA-E has emerged in recent years as a pivotal modulator of adaptive immune responses. Under conditions such as viral or bacterial infections, HLA-E can present pathogen-derived peptides to unconventional, HLA-E-restricted CD8^+^ T cells via their αβ T cell receptor (73, 98, 99). These HLA-E-restricted T cells can bypass classical MHC presentation pathways and mount robust cytotoxic responses, maintaining the detection and clearance of infected cells despite HLA-E-mediated NKG2A^+^ NK cell evasion. While HLA-E-restricted regulatory CD8^+^ T cell responses have been described in human autoimmune disease, such as type 1 diabetes (110, 111), such regulatory phenotypes are not well characterized in the context of viral infections. Instead, most reported HLA-E-restricted T cell responses in viral infection appear to adopt cytotoxic or effector functions (98).

EBV is able to induce an HLA-E-restricted CTL response in the human host (98–100). A recently published study revealed that HLA-E-restricted CD8^+^ T cells recognizing the EBV BZLF1-derived SQAPLPCVL peptide are significantly more frequent in individuals with asymptomatic primary EBV infections. In contrast, low frequencies of these cells were found in patients with symptomatic primary EBV infection (infectious mononucleosis, IM), indicating that these specific T cells play an important part in control of EBV infections. The same study demonstrated that HLA-E*01:03/01:03-expressing cells, compared to HLA-E*01:01/01:01-expressing cells, are associated with especially stable SQAPLPCVL-mediated upregulation of HLA-E, a more pronounced activation and proliferation of HLA-E-restricted, SQAPLPCVL-specific CTLs in response to EBV-infected cells and, subsequently, more efficient inhibition of EBV spread *in vitro* (97).

Besides primary EBV infection, recent studies have also implicated a controversial role of SQAPLPCVL-specific CD8^+^ T cells in the pathogenesis of MS. We have previously reported lower frequencies of these cells in relapsing-remitting (RR) MS patients compared to healthy individuals, especially in individuals with a history of IM (15). Other groups have, however, reported increased frequencies of SQAPLPCVL-specific CD8^+^ T cells in RRMS compared to primary progressive MS patients or healthy controls, potentially indicating a role in RRMS disease pathogenesis or excessive EBV reactivation (112). Together, these observations point toward a multifaceted role of SQAPLPCVL-specific, HLA-E-restricted CD8^+^ T cells in EBV immunity and MS, and their impact may depend on the interplay between host HLA-E genotype, EBV infection history, and disease subtype and stage.

## HCMV-SPECIFIC, HLA-E-RESTRICTED NK CELLS AND MS

Besides CD94/NKG2A, peptide-loaded HLA-E can also bind the CD94/NKG2C receptor, which shares 75% amino acid identity with NKG2A and is similarly expressed on NK cells and CTLs (113, 114). In general, CD94/NKG2C binds peptide-loaded HLA-E molecules with approximately sixfold lower affinity than CD94/NKG2A (115). In addition, CD94/NKG2C recognizes a substantially more restricted peptide repertoire, mainly limited to peptides derived from HLA class I leader sequences in the form of VM(A/P)PRT(L/V)(V/L/I/F)L (115–117).

In contrast to the inhibitory NKG2A, NKG2C functions as an activating receptor. It lacks inhibitory ITIM motifs in its cytoplasmic domain and instead associates with CD94 and the adaptor protein DAP12, the latter containing an activating ITAM motif (118). Engagement of NKG2C with HLA-E/peptide complexes triggers cytolysis of the target cell. NKG2C expression is low in immature NK cells but may increase progressively during maturation, while NKG2A expression declines in parallel. Consequently, mature NK cells typically express either the inhibitory NKG2A or the activating NKG2C, but rarely both (67).

The expression of NKG2C on NK cells is strongly associated with HCMV infection. HCMV infects a large proportion (50%–100%) of the human population and evades T cell recognition by downregulating classical HLA class I molecules. At the same time, it avoids NK cell-mediated lysis by encoding for a UL40-derived peptide that mimics HLA class I leader sequences, thereby promoting HLA-E expression on infected cells and enabling NKG2A-mediated immune evasion. However, HLA-E surface stabilization also permits recognition via the activating CD94/NKG2C receptor and promotes the expansion and activation of virus-specific NKG2C^+^ NK cells. When engaged by the HLA-E/UL40-derived peptide complex, NKG2C stimulates NK cell cytotoxicity and cytokine production, contributing to the control of HCMV infection (30, 119).

NKG2C^+^ NK cells display a distinctive epigenetic profile, with alterations in key transcription factors, signaling adaptors, and surface receptor expression (30, 120–124). These changes are accompanied by specific clonal expansion and a secondary memory response to HCMV reinfection in adaptive NK cell subsets, resembling clonal T cell responses (125, 126). Interestingly, UL40 is a highly polymorphic molecule, and different UL40-derived peptide variants may bind with varying affinities to HLA-E, ultimately affecting the expansion of NKG2C^+^ NK cells (101, 119, 127).

HCMV infection is a major environmental factor shaping immune responses involved in MS. The role of HCMV in MS pathogenesis and progression remains controversial, as it may contribute through different mechanisms to both the development and prevention of MS (20). Observational studies have demonstrated a somewhat lower MS prevalence in HCMV-seropositive individuals and suggested that HCMV infection may provide some protection against the development of MS (14, 128–131). Other studies have reported better clinical outcomes in HCMV-seropositive MS patients (132, 133), and it has been proposed that HCMV-mediated immunomodulation may convey a protective effect in these individuals.

A recent study by our group revealed that specific immune responses directed against a highly conserved peptide sequence within the EBV nuclear antigen 1 (EBNA-1) region, EBNA-1_381-452_, cross-react with distinct CNS-derived proteins and may elicit autoimmune processes through molecular mimicry (134–136). However, potent cytotoxic NKG2C^+^ NK cells can eliminate these autoreactive immune cells via recognition of surface HLA-E on activated, autoreactive B cells, CD8^+^ T cells, and CD4^+^ T cells, thereby potentially providing, to some extent, protection against MS. Consistent with this finding, recent studies have demonstrated that MS patients exhibit less potent NKG2C^+^ NK cell responses than healthy controls. Furthermore, higher frequencies of NKG2C^+^ NK cells in HCMV-seropositive individuals with MS have been associated with lower disability scores and a decreased risk of disability progression, suggesting a potential protective effect mediated through enhanced immunoregulatory functions (137). Mechanistically, impaired NKG2C^+^ NK cell responses in MS have been linked to the absence of HCMV infection, a genetic deletion of the NKG2C receptor—which directly correlates with reduced or absent NKG2C surface levels and decreased frequency of NKG2C^+^ NK cells *in vivo* (138, 139)—or infection with HCMV isolates encoding UL40 peptide variants that induce only weak HLA-E upregulation and limited expansion of NKG2C^+^ NK cells. Ultimately, these factors may reduce NK cell-mediated protection against MS via ineffective control of EBV-related autoimmunity by HCMV-induced NKG2C^+^ NK cells (15). These findings raise the question of whether the induction of high-level NKG2C^+^ NK cells, potentially via therapeutic vaccination with highly potent UL40 peptides or through cellular immunotherapies, could serve as an additional option to limit the pathogenesis and progression of MS.

## HCMV-SPECIFIC, HLA-E-RESTRICTED T CELLS AND MS

While the expansion of NKG2C^+^ cells was initially characterized in the context of NK cell responses, it is now evident that the HCMV–NKG2C–HLA-E axis also extends into adaptive immunity, promoting the expansion of specialized NKG2C^+^ CD8^+^ and CD4^+^ T cell subsets (98, 99, 140–142). Studies have reported that increased NKG2C expression in CD8^+^ T cells correlates with greater disability, as reflected by higher Expanded Disability Status Scale scores in HCMV-seropositive MS patients (143). The pathogenic role of NKG2C T cells in CNS autoimmunity is further supported by post-mortem analyses of MS brain tissue, which show that HLA-E is upregulated on oligodendrocytes in active lesions, where it colocalizes with infiltrating NKG2C^+^ CD4^+^ T cells (144). This spatial proximity strongly suggests direct interactions between HCMV-imprinted T cells and CNS-resident cells via the HLA-E–NKG2C axis. Such interactions may contribute to immune-mediated demyelination and neuronal damage, potentially through bystander killing and amplification of chronic inflammation.

## FUTURE DIRECTIONS

The differential functions mediated by HLA-E in the context of HHV infection depend on the presence of specific viral strains and peptides within the host and host genetic risk factors influencing HLA-E peptide binding, peptide presentation, and interaction with NK and T cells. In light of recent research findings, the collective assessment of these factors, together with the timing of infection and consideration of HHV co-infections, may enable risk stratification for the development and prognosis of HHV-associated acute and chronic neuroinflammatory disorders. HLA-E-associated pathways may also serve as therapeutic targets by enhancing or reducing cytotoxic responses via HLA-E, NK cell-directed or T cell-directed treatments, depending on the underlying pathomechanisms of specific diseases. Future studies will need to focus on the impact of immunomodulatory treatments on these diseases in order to maintain the delicate balance between protective immune activation and excessive, tissue-damaging cytotoxicity. Furthermore, the involvement of HLA-E in the development of neurodegenerative disorders in interplay with HHV infections deserves further investigation, as effective therapeutics for these conditions are limited, and HLA-E-directed interventions could potentially halt progression in these chronic disorders.

## CONCLUSION

HHV infections shape the host immune response over a lifetime, with implications for both acute and chronic neurological disorders. HLA-E is a non-classical MHC class I molecule acting as a ligand for both inhibitory and activating NK and T cell receptors and presenting antigenic peptides to unconventional T cells. Through these roles, HLA-E bridges innate and adaptive immunity, thereby fine-tuning immune activity within the CNS. Under physiological conditions, its low level expression contributes to CNS immune privilege, whereas its upregulation during inflammation may enhance neuroprotection by preventing excessive immune-mediated damage. However, the same mechanisms that preserve tissue integrity can, under certain circumstances, promote pathology. HLA-E surface stabilization via binding of HHV-derived peptides and interaction with the inhibitory CD94/NKG2A receptor on NK and T cells facilitates immune evasion. Conversely, engagement of the activating receptor CD94/NKG2C—particularly in infection-driven or immune-primed contexts—can induce immunoregulatory pathways but may also shift HLA-E’s role toward promoting cytotoxic NKG2C^+^ cell responses, with opposing consequences for neuroinflammatory disease development. Thus, understanding the context-dependent effects of HLA-E may aid in predicting individual risk for neurological disease in HHV-infected individuals and in guiding the design of targeted immunotherapies that protect neural tissue while appropriately modulating neuroinflammation.
